# LONGITUDINAL ASSOCIATIONS BETWEEN FALLS AND DEPRESSIVE SYMPTOMS AMONG CHINESE OLDER ADULTS

**DOI:** 10.1093/geroni/igac059.2712

**Published:** 2022-12-20

**Authors:** Nie Zuoting, Eunjin Lee Tracy, Yan Du, Sha Li, Rumei Yang

**Affiliations:** Nanjing Medical University School of Nursing, Nanjing, Jiangsu, China (People’s Republic); University of Pittsburgh School of Medicine, Pittsburgh, Pennsylvania, United States; UT Health San Antonio, San Antonio, Texas, United States; Nanjing Medical University School of Nursing, Nanjing, Jiangsu, China (People’s Republic); Nanjing Medical University School of Nursing, Nanjing, Jiangsu, China (People’s Republic)

## Abstract

Falls and depressive symptoms are prevalent and costly to Chinese older adults. Although falls and depressive symptoms are frequently interrelated, previous studies were mainly based on cross-sectional data, and the nature of interrelationships between them were not well understood. In this study, we aimed to examine the longitudinal bidirectional relationship between falls and depressive symptoms among Chinese older adults, and whether this bidirectional relationship is different for males and females, given that females are found to suffer from falls and depressive symptoms significantly more than males. Older adults aged 60 years+ who completed all three waves of data from the China Health and Retirement Longitudinal Study on falls and depressive symptoms between 2011 (wave 1), 2013 (wave 2), and 2015 (wave 3) were included in the current analysis (Nf2,203). Data were analyzed using random intercept multilevel models that adjusted for demographic information (e.g., age, education, and marital status). Overall, there is a relatively stable rates of comorbid falls and depression (about 10%) over time among Chinese older adults. Significant bidirectional associations at between-person and within-person levels were observed between depressive symptoms and falls over time, with greater depressive symptoms associated with higher risk for falls and vice versa. However, such associations were not different between males and females. Taken together, these findings indicate the importance of addressing both depression and falls at the same time to prevent falls in the clinical practice for both males and females.

